# Facile fabrication of polystyrene particles/graphene composites for improved dielectric and thermal properties

**DOI:** 10.1080/15685551.2022.2162282

**Published:** 2022-12-28

**Authors:** Wei Deng, Guoan Li, Wanyu Li, Meng Yang, Weiwei Cui

**Affiliations:** a School of Material Science and Chemical Engineering, Harbin University of Science and Technology, Harbin, Heilongjiang, China; b Key Laboratory of Engineering Dielectric and Its Application, Ministry of Education, Harbin University of Science and Technology, Harbin, Heilongjiang, China

**Keywords:** Graphene, polystyrene particles, dielectric properties, breakdown strength, thermal stability

## Abstract

In this paper, polystyrene (PS)-based reduced graphene oxide (rGO) composites were prepared by mixing PS latex particles with graphene oxide (GO) and the following in-situ reduction. The structure and morphology of PS/rGO composites were characterized, and the effects of rGO content on the dielectric properties as well as thermal stability of PS/rGO composites were investigated. Results showed that rGO sheets armoured on the surface of PS particles and exhibited well dispersion in the PS matrix after hot compression. The introduction of rGO improved the dielectric properties of the composites remarkably. When rGO content was 0.12 vol%, the dielectric permittivity and breakdown strength of PS/rGO arrived at 6.3 at10^2^ Hz and 107 kV/mm, with 50% and 35.4% enhancement compared to the pristine PS. Furthermore, PS/rGO presented better thermal stability than the pristine PS, but the overlapping of rGO sheets in PS matrix induced the instability of dielectric loss with frequency.

## 1. Introduction

With the continuous development of electronic products towards miniaturization and multifunction, intensive research has been focused on the dielectric materials with good comprehensive properties. Compared with inorganic ceramics, polymer has good flexibility, processability and low cost, but extremely low dielectric constant limits its applications as dielectrics. Introducing ceramic or conductive materials as fillers is the common strategy to improve the dielectric properties of polymers. For polymer-ceramic composites, the high dielectric constant usually achieves through adding lots of ceramic fillers, which inevitably leads to the sacrifice of mechanical properties and processability [1–4]. While conductive fillers, such as metal particles, carbon black, carbon nanotube, graphene and graphene derivatives, have been impressive, as they can significantly enhance the dielectric constant of polymer-based materials at extremely small loading based on the construction of percolation system [5–8].

Graphene, a two-dimensional layered structure of sp^2^ bonded carbon atoms, has attracted tremendous attention due to its giant electron mobility, high thermal conductivity, excellent mechanical strength, and ultrahigh specific surface area, and emerged as promising nanofiller for the fabrication of advanced polymer-based dielectric composites, providing great potential applications in electron and electrical equipment [9]. Many methods including melt blending, solution mixing, and in-situ polymerization have been proposed to develop polymer/graphene composite dielectric materials [10–12]. However, the high hydrophobicity and strong π-π interaction result in the aggregation, restack and poor dispersion of graphene nanosheets in most solvents and polymers, which impose restrictions on the contribution of graphene to the performances of the composites. As a derivative of graphene, graphene oxide (GO) contains hydroxyl and epoxy groups on its basal plane and carboxylic acid groups at the edge, which is beneficial to its dispersion and functionalization, thus improving the compatibility with polymers. After the reduction reaction, GO can be transformed into reduced graphene oxide (rGO) and subsequently acts as conductive fillers, so GO is widely used as a precursor for preparation of polymer/graphene compositess [13–15].

For polymer-based graphene composites, the dispersion of graphene directly correlates with its effectiveness for improving the macroscopic properties [16,17]. As for now, the predominant method to obtain polymer-based graphene composites with high dielectric constant is modifying and orienting graphene [8,18–20]. Polystyrene (PS) is a highly commercialized thermoplastic material with the features of good optical transparency, low price, mechanical properties, and excellent electric insulation. Moreover, all sorts of polymerization methods are suitable for the synthesis of PS, and the morphology of PS is prone to be designed and controlled. However, the dielectric constant of PS is as low as about 2.8 at 100 Hz, limiting its application in the electronics area. In order to improve the dielectric properties of PS, several studies have appeared on enhancing the dispersity of graphene in PS matrix. For example, Wu et al. reported the preparation of PS composite with TiO_2_ modified graphene hybrid sheets, and the resultant composite exhibits a very high permittivity of 1741 at 100 Hz with 10.9 vol% graphene-TiO_2_ hybrid sheets [21]. Zhang et al. fabricated PS composites with PS-grafted rGO (rGO-PS) as fillers, and the dielectric constant of the PS/rGO-PS composites increased to 395 at 100 Hz, accompanied with the dielectric loss of 0.45 [22].

In this study, PS particles/rGO composites were prepared by latex blending of PS particles with GO and the following in-situ reduction, then PS/rGO composite sheets were obtained by hot compression molding process. The PS particles effectively prevent the aggregation of rGO sheets, and endow the PS/rGO composite sheets with simultaneously increased dielectric constant and breakdown strength. This work provides a facile method and new insight to prepare polymer composites with improved dielectric properties from the view of adjusting the polymer matrix morphology instead of modifying graphene.

## 2. Experimental

### 2.1 Materials

Styrene (St), methylacrylic acid (MAA) and ammonium persulfate (APS) were purchased from Tianjin Guangfu Fine Chemical Research Institute, China. St and MAA were purified by distillation under reduced pressure, and APS was purified by recrystallization twice before use. Graphite powder and hydrazine (80%) were obtained from Tianjin Qibang Chemical products sales Co. Ltd., China and used as received.

### 2.2 Preparation of PS latex particles

PS latex particles were prepared by batch surfactant-free emulsion polymerization in a 250 ml four-necked round bottom flask equipped with a reflux condenser, a mechanical stirrer and a thermometer in nitrogen atmosphere at 70°C for 7 h. First, 19.33 g of St and 0.67 g of MAA were dispersed in 100 g of H_2_O. After stirring for 30 min, 0.336 g of APS dissolved in 25 g of H_2_O was introduced at the beginning, 2 h and 4 h of polymerization with a ratio of 12:8:5.

### 2.3 Preparation of PS/rGO composites

**Figure 1.**
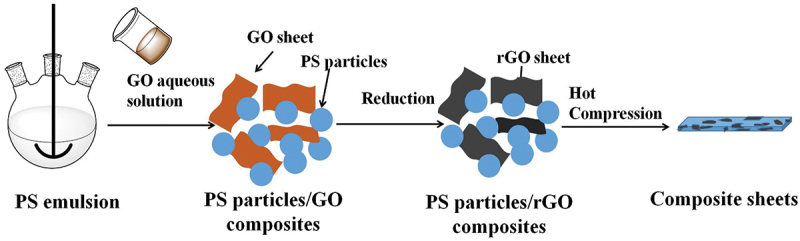
Schematic for the preparation of PS particles/rGO composites and corresponding composite sheets.

The PS particles/rGO composites were prepared by mixing the PS latex particles with the GO aqueous dispersion and the following reduction of GO in the presence of hydrazine, as shown in Figure 1. Firstly, GO was prepared from graphite powder by modified Hummers method [23] and dispersed into water with a concentration of 1 mg/ml, then a certain amount of GO dispersion was mixed with 30 g of PS emulsion. After sonication for 30 min, hydrazine with an equivalent amount of GO was added, and the system was heated to 90°C for 2 h under vigorous stirring to obtain PS particles/rGO composites.

The reduction yield of GO to rGO was obtained by a control experiment without PS particles. After adding hydrazine into the GO aqueous dispersion, some black solids gradually precipitated which were collected and dried to obtain the rGO nanosheets. The reduction yield (*R_y_*) of ~0.5 was obtained in our experiments. Then, the weight fraction of rGO (*W_f_*) in the PS/rGO composites can be calculated by the following equation:

$$W_{f}=\frac{m_{GO}\times R_{y}}{m_{GO}\times R_{y}+m_{PS}}\times 100\%$$

where *m_GO_* and *m_PS_* represent the mass of GO and PS particles, respectively. At last, the volume fraction of rGO (*V_f_*) in the PS/rGO composites can be transformed from *W_f_* by the following equation:

$$V_{f}=\frac{W_{f}/\rho_{rGO}}{W_{f}/\rho_{rGO}+\left(1-W_{f}\right)/\rho_{PS}}\times 100\%$$

where *ρ_rGO_* and *ρ_PS_* represent the density values of rGO (2.26 g/cm^3^) and PS particles (1.06 g/cm^3^), respectively.

When the usage of GO was 0.5, 1.0, 2.0, 3.0, 4.0 and 5.0 wt% to dried PS particles, the corresponding volume fraction of rGO was 0.12, 0.23, 0.46, 0.72, 0.96 and 1.22 vol%. After cooling down to room temperature, the products were filtered and dried, then the resultant composite powders were compression molded at 200°C under 2 MPa for 15 min to obtain PS/rGO composite sheets.

### 2.4 Characterization

FTIR was recorded using a Nicolet iS10 spectrometer (USA) with KBr pellet. XRD was carried out with a Rigaku D/MAX-2500 (Japan) using Cu Kα radiation source. SEM was conducted with a Hitachi SU8020 microscope (Japan) at an accelerating voltage of 1 kV and a FEI sirion200 microscope (Holland) at an accelerating voltage of 20 kV to observe the surface and fracture surface of the composites, respectively. The diameter distribution of particles was characterized by Malvern Zetasizer 3000HSA (UK). The Dielectric properties of the composites were determined by broad dielectric tester (HIOKI3532-50 LCR meter, Japan) in the frequency range of 10^2^–10^7^ Hz at room temperature. The breakdown strength was measured by HT-100 withstand voltage tester (China) and the voltage was continuously increased at the rate of 1kV/s until the sample broken down. The thermal gravimetric analysis (TGA) was carried out by using an STA 449F3 simultaneous thermal analyzer (Germany) at a heating rate of 10°C/min from 40°C to 550°C in nitrogen atmosphere.

## 3. Results and discussion

### 3.1 Microstructure and morphology of PS/rGO composites

**Figure 2.**
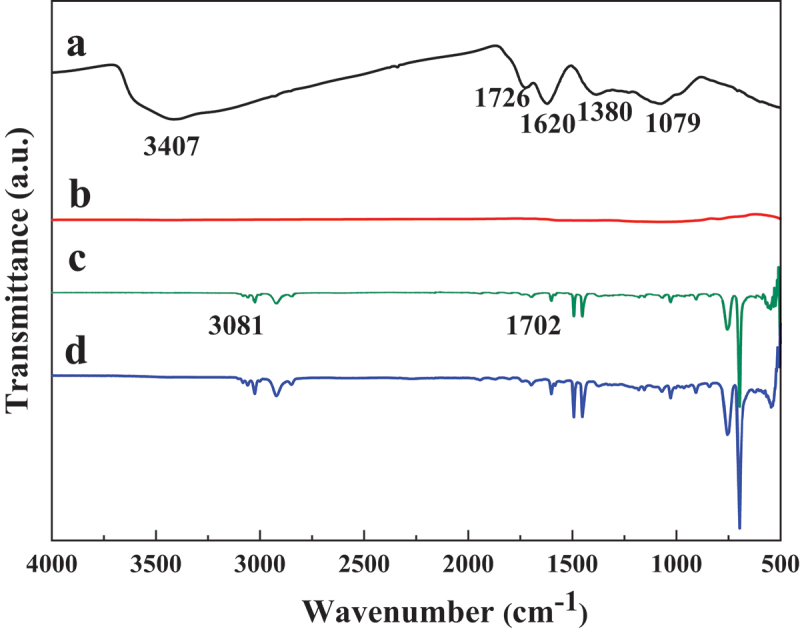
FTIR spectra of (a) GO, (b) rGO, (c) PS particles and (d) PS particles/rGO composites.

The FTIR spectra of GO, rGO, PS particles and PS particles/rGO composites containing 1.22 vol% of rGO are shown in Figure 2. In the spectrum of GO, the broad peak at 3407 cm^−1^ is assigned to the O-H stretching vibration. The peaks at 1726 cm^−1^, 1380 cm^−1^ and 1079 cm^−1^ are related to the carbonyls (C = O) stretching vibration, O-H deformation vibration and C-O stretching vibration, respectively. The absorption at 1620 cm^−1^ should be attributed to the skeletal vibrations of unoxidized graphitic domains [24,25]. These observations suggest that oxygen-containing groups have been introduced onto the GO sheets. During the preparation of PS latex particles via emulsifier-free polymerization, MAA was used to avoid the coalescence of latex particles. In this case, besides the characteristic bands of PS at 1450–1600 cm^−1^ and 690–760 cm^−1^, the adsorption of O-H at 3081 cm^−1^ and C = O at 1702 cm^−1^ appears in spectrum of PS particles, indicating the successful fabrication of carboxyl-functionalized PS particles. In a control experiment without usage of PS latex particles, GO was reduced by equivalent amount of hydrazine. It can be seen that most oxygen-containing groups on GO are effectively removed after reduction and rGO has no obvious adsorption in the FTIR spectrum. Therefore, there is no obvious difference between the FTIR results of the pristine PS particles and the PS particles/rGO composites.

**Figure 3.**
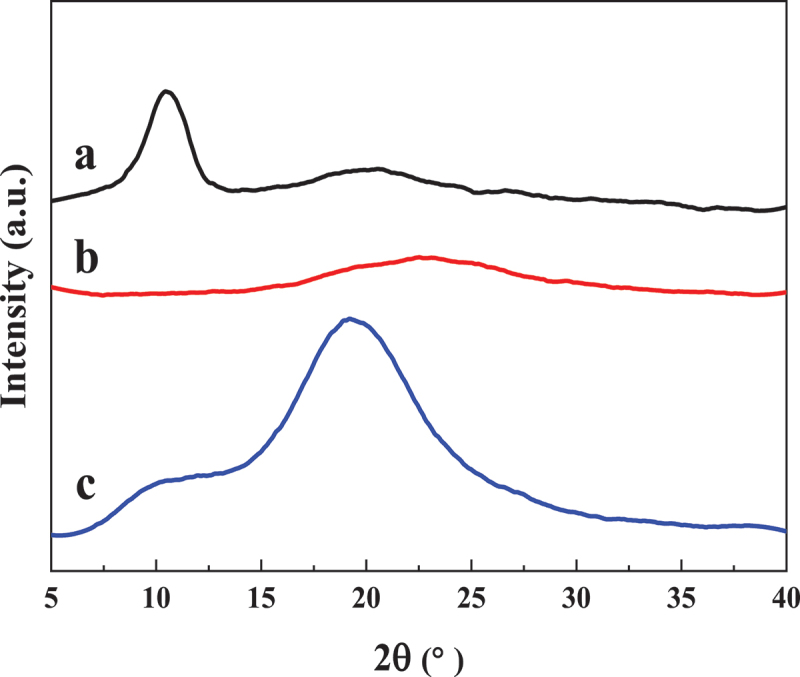
XRD patterns of (a) GO, (b) rGO and (c) PS/rGO composites.

The XRD patterns of GO, rGO and PS particles/rGO composites containing 1.22 vol% of rGO are presented in Figure 3. The strong peak at 2θ = 10.6° corresponds to the basal plane (001) of GO. After reduction by hydrazine, the resultant rGO displayed broad peak at 2θ = 22.3°, which is associate with the (002) diffraction peak of the graphitic structure, indicating that the aggregation of single-layer and few-layer rGO nanosheets occurs after the chemical reduction [26]. However, the aforementioned peaks are not detected and only the amorphous peak of PS at 2θ = 19.4° is obvious in the XRD profile of PS particles/rGO composites, suggesting that the presence of PS latex particles inhibits the aggregation of rGO effectively.

**Figure 4.**
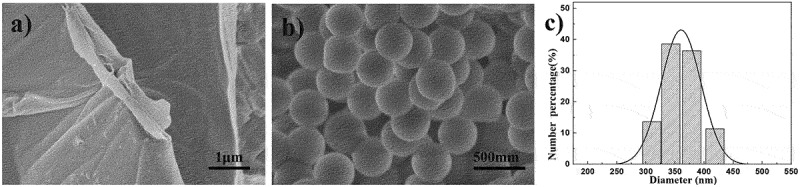
SEM image of (a) GO and (b) PS particles, and (c) diameter distribution of PS particles.

**Figure 5.**
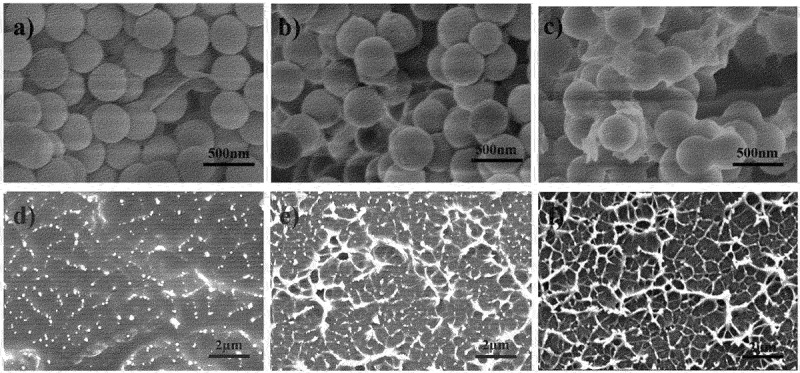
SEM images of PS/rGO composites before compression molding (a) 0.12 vol% rGO, (b) 0.46 vol% rGO and (c) 1.22 vol% rGO, and the corresponding cross sectional SEM images (d), (e), (f) after compression molding.

The morphologies of GO, PS particles, PS particles/rGO composites and PS/rGO composite sheets are presented in Figure 4 and Figure 5. Apparently, GO exhibits flaky structure with wrinkles (Figure 4(a)) and PS particles show uniform spherical (Figure 4(b)). Dynamic light scattering analyzes reveal that the average hydrodynamic diameter of PS particles is 360 nm (Figure 4(c)). It can be seen from Figure 5 that rGO sheets well disperse and incorporate into the polymer matrix. The intercalation of PS particles is beneficial to peeling the rGO sheets, and the adhesion between rGO sheets and PS particles becomes clearer with the increasing of rGO content, which can be attributed to the large surface of graphene sheets, PS particles and the π-π stacking interactions between graphene and phenyl group in polystyrene. Moreover, no obvious agglomeration of graphene sheets is found in the fracture surfaces of PS particles/rGO composite sheets, and the protruding network-like stacks of rGO sheets clearly increase with the increase of rGO content (Figure 5(d)-(f)), even forming fully connected network when rGO content is 1.22 vol%.

### 3.2 Dielectric properties of PS/rGO composites

**Figure 6.**
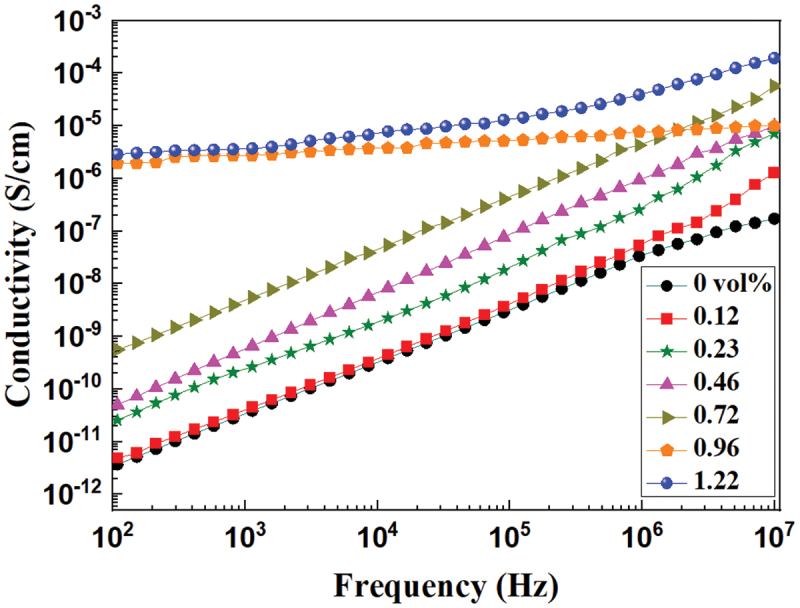
Frequency dependence of conductivity of the PS/rGO composites with different rGO content at room temperature.

The frequency dependence of the alternating current (AC) conductivity of PS/rGO composites containing various rGO contents is shown in Figure 6. At low rGO volume fraction (≤0.72 vol%), the AC conductivity almost linearly increases with increasing frequency, indicating their insulating nature. Along with the volume fraction of rGO growing to 0.96 vol%, the conductivity increases abruptly to higher than 10^−6^ S/cm and only slightly changes with the frequency in the range of 10^2^ to 10^7^ Hz, indicating the occurrence of percolation phenomenon.

**Figure 7.**
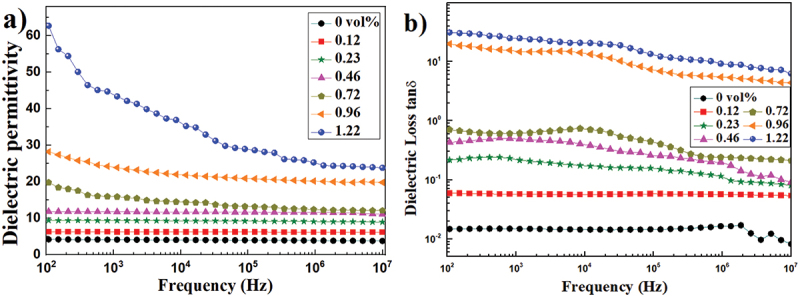
Frequency dependence of (a) dielectric permittivity and (b) loss factor of the PS/rGO composites with different rGO content at room temperature.

Figure 7 shows the study of dielectric permittivity (ε_r_) and dielectric loss factor (tanδ) of PS/rGO composites as a function of frequency at room temperature. The dielectric permittivity of pristine PS slightly decreases from 4.21 to 3.77 as the frequency increased from 10^2^ to 10^7^ Hz. The dielectric permittivity of PS/rGO composites increases with increasing rGO content through the entire frequency range and exhibits a typical percolation transition behavior. At low rGO volume fraction (≤0.72 vol%), the conductive rGO nanosheets are isolated by the insulating PS particles to form many micro-capacitor structures, and the electrons accumulate at such interfaces under applied electric field, leading to interfacial polarization, that is Maxwell-Wagner-Sillars polarization, which is the main reason for the improvement in dielectric permittivity of PS/rGO composites at low frequency. The dielectric permittivity at 10^2^ Hz goes from 6.2 at loading of 0.12 vol % rGO to 20 at loading of 0.72 vol% rGO, which increases by nearly 5 times compared with the pristine PS. As the frequency increases, dipoles fail to keep up with the transformation of the applied electric field, resulting in the depression of dielectric permittivity [27]. When the content of rGO reaches 0.96 vol%, the composites change from insulator to semiconductor, and the significant increase in dielectric permittivity arises from the well establishment of the tunneling percolation network [28], which is accordance with the conductive path observed in Figure 5(f).

The dielectric loss factor also increases with the incorporation of rGO nanosheets, and dramatically goes up to very high value when the content of rGO is 0.96 vol%. For the composites containing conductive fillers, percolation theory can explain the aforementioned phenomena. Below the percolation threshold, the dielectric loss mainly results from interfacial polarization and space charge, while above it, high content of rGO nanosheets may construct long conductive pathways, and the electric conductive loss caused by leakage current comes into prominence, causing the significant increase in the dielectric loss factor of polymer-based graphene composites [29].

### 3.3 Breakdown strength of PS/rGO composites

**Figure 8.**
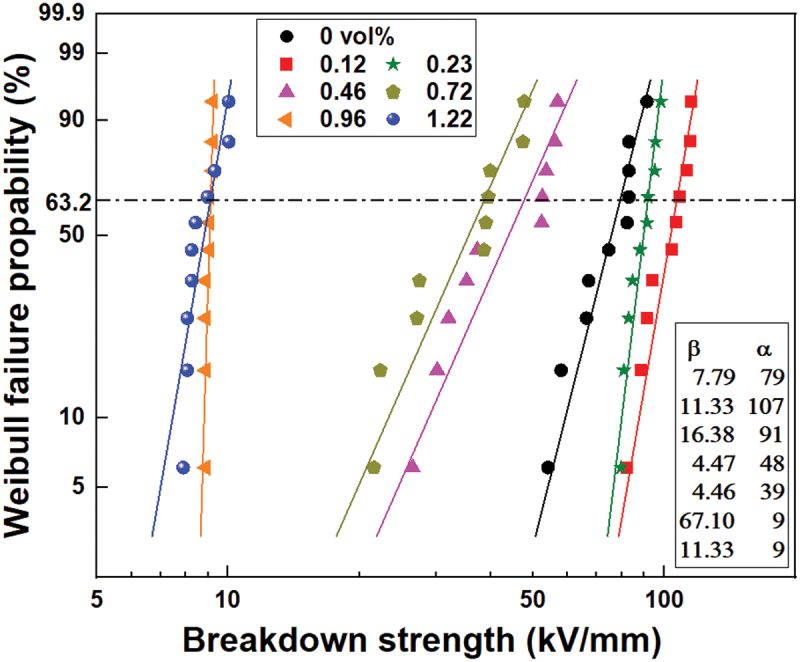
Weibull plot for DC breakdown strength of the PS/rGO composites with different rGO content at room temperature.

The two Weibull plot for DC breakdown strength of pristine PS and PS/rGO composites with different content of rGO is displayed in Figure 8. Herein, α is the scale parameter that represents the Weibull DC breakdown strength at the cumulative breakdown probability of 63.2%, and β is the shape parameter that reflects the scatter degree of breakdown data. rGO has high thermal conductivity and may reduce the thermal breakdown possibility of composites. On the other hand, the interfacial interaction between rGO and PS increases the potential barrier of the electron transition so as to restrict the movement of electrons. Consequently, the breakdown strength of PS/rGO composites increases first with the increasing of rGO content and reaches its maximum value of 107 kV/mm at rGO 0.12 vol%, which is 35.4% higher than that of pristine PS. As the content of rGO is higher than 0.46 vol%, the agglomeration effect and leakage current can bring about obvious depression of the breakdown strength [30].

### 3.4 Thermal stability of PS/rGO composites

**Figure 9.**
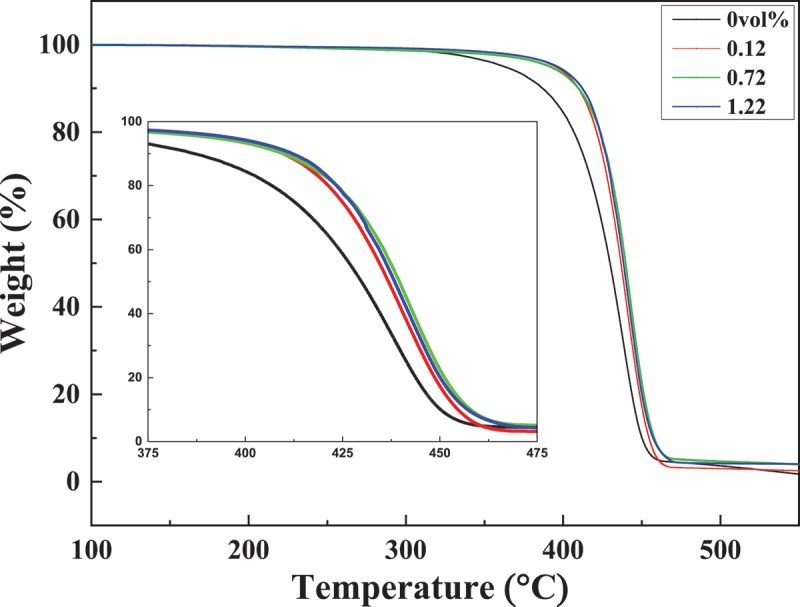
TGA curves of the PS/rGO composites with different rGO content.

The thermal stability of pristine PS and PS/rGO composites are shown in Figure 9. It is observed that the thermal decomposition of PS occurs in a single step. The initial slight weight loss of pristine PS is attributed to the elimination of oxygen-containing functional groups originated from the copolymerization monomer MAA, and the decomposition temperature at 10% weight loss is 386°C. Then the weight loss steps into rapid decline, which corresponds to β-depolymerization of polystyrene [31]. All the PS/rGO composites show similar thermal decomposition behavior to the pristine PS particles, but the decomposition temperature at 10% weight loss rises to 410°C, and the weight of residue increases with the increasing of rGO content. Apparently, PS/rGO composites possess better thermal stability than pristine PS particles. This is probably due to the inherent excellent thermal stability of rGO, the strong interfacial interaction between rGO nanosheets and PS particles, and the steric effect of hindering the thermal motion of polymer chains [32].

## 4. Conclusion

Flaky GO has been successfully prepared and added into PS latex particles to obtain PS particles/rGO composites by in-situ reduction. The micro-structure, dielectric and thermal properties of the composites with different content of rGO are investigated. The insertion of PS particles into rGO sheets and the strong interfacial interaction between the rGO and PS particles allowed rGO to disperse in PS matrix uniformly. As the content of rGO increases, a typical percolation transition occurs and leads to the rapid rise in the conductivity, dielectric permittivity, and dielectric loss of the PS/rGO composites. The PS/rGO composites containing 0.72 vol% rGO maintain the insulation nature with a dielectric permittivity of 20 at 10^2^ Hz. Compared with the pristine PS, the DC breakdown strength of the PS/rGO composites increases by 35.4% to 107 kV/mm when the content of rGO is 0.12 vol%. Furthermore, the introduction of rGO improves the thermal stability of PS/rGO composites.
